# Bullying and Victimization in Overweight and Obese Outpatient Children and Adolescents: An Italian Multicentric Study

**DOI:** 10.1371/journal.pone.0142715

**Published:** 2015-11-25

**Authors:** Dario Bacchini, Maria Rosaria Licenziati, Alessandra Garrasi, Nicola Corciulo, Daniela Driul, Rita Tanas, Perla Maria Fiumani, Elena Di Pietro, Sabino Pesce, Antonino Crinò, Giulio Maltoni, Lorenzo Iughetti, Alessandro Sartorio, Manuela Deiana, Francesca Lombardi, Giuliana Valerio

**Affiliations:** 1 Dipartimento di Psicologia, Seconda Università di Napoli, Caserta, Italy; 2 Dipartimento di Pediatria Specialistica e Sistematica, AORN Santobono Pausilipon Annunziata, Napoli, Italy; 3 Servizio di Dietologia Clinica, Ospedale Pediatrico Bambino Gesù IRCCS, Roma, Italy; 4 Centro di Prevenzione Diagnosi e Cura dell’Obesità in Età Evolutiva U.O. Pediatria, Presidio Ospedaliero di Gallipoli, Lecce, Italy; 5 SOC Clinica Pediatrica, Azienda Ospedaliero-Universitaria, Udine, Italy; 6 U.O. di Pediatria, Azienda Ospedaliero-Universitaria S. Anna, Ferrara, Italy; 7 Dipartimento della Donna, del Bambino e di Chirurgia Generale e Specialistica, Seconda Università di Napoli, Napoli, Italy; 8 UO di Pediatria, Ospedale S. Liberatore, Atri, Italy; 9 UOC di Malattie Metaboliche ed Endocrinologia Pediatrica A.O.U. Policlinico di Bari, Bari, Italy; 10 U.O.S. di Patologia Endocrina Autoimmune, Ospedale Pediatrico Bambino Gesù IRCCS, Palidoro, Roma, Italy; 11 Dipartimento di Scienze Mediche e Chirurgiche, Unità Operativa di Pediatria, Programma di Endocrinologia Pediatrica, Policlinico Universitario S. Orsola Malpighi, Bologna, Italy; 12 Dipartimento di Scienze Mediche e Chirurgiche Materno-Infantili e dell’Adulto, Università di Modena e Reggio Emilia, Modena, Italy; 13 Istituto Auxologico Italiano, IRCCS, Divisione di Auxologia e Malattie Metaboliche, Verbania, Italy; 14 Clinica Pediatrica, Ospedale F. Del Ponte, Varese, Italy; 15 U.O.C. di Pediatria, Ospedale Maggiore, Ausl di Bologna, Bologna, Italy; 16 Dipartimento di Scienze Motorie e del Benessere, Università degli Studi di Napoli Parthenope, Napoli, Italy; University of Bath, UNITED KINGDOM

## Abstract

**Objective:**

Being overweight or obese is one of the most common reasons that children and adolescents are teased at school. We carried out a study in order to investigate: i) the relation between weight status and school bullying and ii) the relation between weight status categories and types of victimization and bullying in an outpatient sample of Italian children and adolescents with different degrees of overweight from minimal overweight up to severe obesity.

**Participants/Methods:**

Nine-hundred-forty-seven outpatient children and adolescents (age range 6.0–14.0 years) were recruited in 14 hospitals distributed over the country of Italy. The participants were classified as normal-weight (N = 129), overweight (N = 126), moderately obese (N = 568), and severely obese (N = 124). The nature and extent of verbal, physical and relational bullying and victimization were assessed with an adapted version of the revised Olweus bully-victim questionnaire. Each participant was coded as bully, victim, bully-victim, or not involved.

**Results:**

Normal-weight and overweight participants were less involved in bullying than obese participants; severely obese males were more involved in the double role of bully and victim. Severely obese children and adolescents suffered not only from verbal victimization but also from physical victimization and exclusion from group activities. Weight status categories were not directly related to bullying behaviour; however severely obese males perpetrated more bullying behaviour compared to severely obese females.

**Conclusions:**

Obesity and bullying among children and adolescents are of ongoing concern worldwide and may be closely related. Common strategies of intervention are needed to cope with these two social health challenges.

## Introduction

Obesity and bullying among children and adolescents are of ongoing concern worldwide [1–4].

Is there a linkage between these two social health challenges? Even though the negative stigmatization towards obese individuals has been widely documented [5–8], the link between bullying and obesity is a research field that is relatively less explored and has begun to receive focused attention only in the last decade [9,10].

Bullying is a specific type of aggression involving intentional, repetitive abuses against peers, aimed at causing harm to the victim operating within an imbalance of power between bullies and victims [11,12]. Bullying can be either direct or indirect and can occur through a variety of actions including physical contact (hitting, pushing), verbal abuse (teasing, name calling), spreading rumors and social exclusion. Bullying is widespread throughout the world, involving young children and adolescents, as well as boys and girls; its negative consequences on child adjustment have been widely demonstrated [13–18].

Being overweight or obese is one of the most common reasons that children and adolescents are teased or bullied at school [5–7,19–21]; in fact, weight-based victimization occurs at school more often than victimization due to race, religion or disability [7,22–25]. Weight-based teasing in overweight or obese children and adolescents may contribute to negative emotional consequences [26], academic failure [21], and peer rejection [7]. Moreover, the risk of depression and low self-esteem in obese children victims of bullying is substantial after controlling for BMI and other variables such as age, gender and duration of obesity, indicating that the psychological *sequelae* are a consequence of victimization and not simply of weight status [27,28].

Only few studies have specifically investigated which types of victimization are highly related to weight status categories, yielding contrasting results. Wang et al. [29] found that overweight boys and obese girls were more likely to be verbally victimized, while no differences were found in the other types of victimization. More recently, Puhl et al. [30], examining in-depth the weight-based experiences of victimization in weight loss treatment–seeking youths, found that verbal teasing was the most frequent type of victimization reported by obese adolescents, followed by relational aggression, cyberbullying and physical aggression. Differently, Janssen et al. [6] observed that overweight and obese youths had greater relative odds of being victims of both relational and overt victimization compared with age-matched normal-weight participants, while an association between overweight and physical victimization was found only in females.

The few studies investigating the involvement of overweight and obese children as perpetrators reported controversial results [31,32]. Janssen et al. [6] found associations between BMI categories and bullying only in 15- to 16-year-old overweight and obese adolescents but not in 11- to 14-year-old teens. We are not aware of any study that examined the involvement of overweight or obese children in the combined role of bully-victim.

Gender seems to have a crucial role, too. In fact, several studies found that overweight and obese youths of both genders are at increased risk of peer victimization [32], while others [5] found that obese boys were significantly more likely to be bullies and victims, whereas obese girls were only victims.

Against this background, the present study was aimed at adding a new contribution to the literature about: i) the relation between weight status categories and bullying and/or victimization in a sample of the Italian paediatric population with different degrees of overweight from minimally overweight up to severe obesity; and, ii) the association between weight status categories and types of victimization and bullying.

## Materials and Methods

### Participants

Nine hundred forty-seven children (age range 6.0–14.0 years) participated in this cross-sectional study. They were recruited in 14 hospitals distributed across northern (Mantova, Udine, Varese, Verbania), central (Atri, Bologna, Modena, Parma, Roma: 2 sites) and southern Italy (Bari, Gallipoli, Naples: 2 sites). The participants were consecutively enrolled in numbers weighted to each pediatric service population. A first setting of recruitment was constituted by outpatient clinics for the treatment of childhood overweight and obesity; a second setting was constituted by hospital ambulatories attended by children for preventive health care or minor complaints (recovery after infectious acute illness, minor medical procedures, dental services, or dermatology services).

The participants in the first setting were selected according to the following inclusion criteria: i) being overweight or obese (see below); ii) age 6–14 years; iii) no other independent pathology associated with weight status; iv) regularly attending school (1^st^ to 8^th^ grade). Eight hundred eighteen participants satisfying the above-mentioned criteria were enrolled, constituting the overweight and obese sample. The participants in the second setting were selected according to the following inclusion criteria: i) normal weight; ii) age 6–14 years; iii) no previous or active pathology at the time of interview; iv) regularly attending school. One hundred twenty-nine participants satisfying the above-mentioned criteria were enrolled, constituting the normal-weight sample, while eight underweight participants were excluded from the study because they could also be at risk of victimization. All participants were consecutively recruited between November 2011 and May 2012. The study was approved by the Ethical Committee of the Department of Psychology of the Second University of Naples and by the Childhood Obesity Group Review Board of the Italian Society of Pediatric Endocrinology and Diabetology. Written informed consent was obtained from all participants and/or their parents or legal guardians in accordance with the revised version of the Helsinki Declaration regarding research involving human subjects.

### Measures

#### Anthropometric and sociodemographic measures

Body weight was determined to the nearest 0.1 kg on accurate and properly calibrated standard beam scales, in minimal underclothes and no shoes. Height was measured to the nearest 0.5 cm on standardized, wall-mounted height boards according to standardized procedures. The BMI was calculated as weight divided by square of height (kg/m^2^).

The BMI standard deviation score (BMI-SDS), calculated using the CDC standards [33] was employed to define normal-weight (NW) (BMI-SDS between -1 and +1); overweight (OW) (BMI-SDS between 1.01 and 1.63), moderate obesity (Mod-OB) (SDS-BMI between 1.64 and 2.5); and severe obesity (Sev-OB) (BMI-SDS >2.5).

Information about ethnic group (Italians vs other ethnic groups) and parents’ educational level (number of school years) were collected.

#### Bullying involvement

We administered a modified version of the “revised Olweus Bully/Victim Questionnaire” [34], adapted and widely used in Italy [35,36]. Two items aimed to specifically detect weight-based victimization, namely “have you ever been teased about your weight status” and “have you ever been excluded from sports activities by your peers?” were inserted on an *ad hoc* basis. Overall, 11 types of bullying were presented in the questionnaire: five concerned direct bullying, either verbal or physical (teasing about physical appearance; teasing for other reasons; verbal offenses; physical aggression, e.g. being hit or kicked; being threatened), six concerned indirect or relational bullying (ignored by peers; stealing money or other things; spreading rumors; exclusion from sports activities; exclusion from other group activities; exclusion from parties). Questionnaires were administered in a separate room by a trained assistant who followed the same administration protocol shared among the participating centers. The assistant had a briefing with the participant in order to ensure that the key concepts included in the standard definition of bullying, such as the intention to harm the victim, the repetitive nature of bullying, and the imbalance in power between the victim and the perpetrator(s), were clearly understood. The participants were asked to fill out the questions: “how often have you been bullied at school in the past couple of months?”; “how often have you taken part in bullying another student(s) at school in the past couple of months?” using a five point scale (“never involved”, “only once or twice”, “2 or 3 times a month”, “about once a week”, “several times a week”). The questionnaire was self-administered but the assistant remained in the room to provide support, if requested by the participants. Only if the participants had limited reading skills (e.g. younger children) the assistant read the single items aloud. No protocol deviations were registered during the data collection. To code involvement in bullying or victimization, both categorical and dimensional approaches were used. In the categorical approach, individuals were classified as bullies, victims and bully-victims if they exceeded a cut-off of two or three episodes a month or more (respectively as bully, victim or both) in an established period (the last two months), according to standard procedures [34]. So, occasional involvement as a bully and/or victim was not considered. In the dimensional approach, a continuous score of single bullying behavior or victimization was considered by assigning point values to each item from 1 (never involved) to 5 (several times a week). In addition, a total score of bullying or victimization was computed by summing the scores of all items of bullying and victimization respectively. Higher scores indicate a greater frequency of engaging in bullying behavior or victimization.

### Statistical analyses

The Statistical Package of Social Sciences (SPSS) for Windows software program release 21.0 was used. The data were expressed as observed frequencies and percentages, mean or adjusted mean, and standard deviation (SD) or standard error (SE) when appropriate. A p value <0.05 was considered significant.

The chi square test was performed to test the association between bullying roles and weight status categories, a standardized residuals value ±2 was considered as a significant measure of the strength of the difference between the observed and expected values.

Because the continuous scores of bullying or victimization were not normally distributed, we normalized all scores using a log transformation; for clarity of interpretation, the results are expressed in the tables as untransformed values. Subsequently, ANCOVA tests 4 x 2 (weight status categories by gender) were performed in order to compare the association between the scores of specific types of bullying or victimization, the weight-status categories and gender, removing the effects of confounding variables. Therefore weight status and gender were the independent variables, while age, ethnicity and parents’ education level were covariates/control variables. Post-hoc analyses (Sidak’s post hoc comparison test) were performed for sub-group comparisons.

## Results

### Bullying roles and weight status

The sample was represented by 129 NW participants (males 38.8%, mean age 11.6 years), 126 OW (males 47.6%; mean age11.4 years), 568 with Mod-OB (males 54.8%; mean age10.9 years), and 124 with Sev-OB (males 55.4%; mean age10.1 years).

Preliminarily, the answers to the bully-victim questionnaire were analyzed in the whole sample following the categorical standard procedure: 7.2% children were coded as bullies, 27.5% as victims, 33.3% as bully-victims, and 32.1% as not-involved. Gender effect was not significant (χ^2^ = 1.37; 3; p = .71), while there was a main effect of age (F = 12.11; 3, 943; p < .0001), because bullies were significantly older [mean age = 12.01 (SD = 1.95) years] than those who were not involved [11.12 (2.02) years], bully-victims [10.84 (1.96) years] and victims [10.50 (1.87) years].

Subsequently, a bivariate cross tabulation between bullying roles and weight-status was performed. The distribution of observed frequencies and percentages related to the cross tabulation between weight status and bullying roles is reported in Table 1. The chi square test was significant (χ^2^ = 35.25; 10; p < .0001). We found significant interactions in the following cases: bullies and not involved were significantly more frequent, whereas bully-victims were less frequent among NW children; bully-victims were more frequent whereas not involved participants were less frequent among Sev-OB children.

**Table 1:** Bullying roles by weight status.

	NW (n = 129)	OW (n = 126)	Mod-OB (n = 568)	Sev-OB (n = 124)
	f (%); SR	f (%); SR	f (%); SR	f (%); SR
Bullies (n = 67)	16 (12.4%); 2.5*	11 (8.7%); 0.8	36 (6.3%); -1.1	4 (3.2%); -1.8
Victims (n = 260)	29 (22.5%); -1.4	25 (19.8%); -1.9	169 (29.9%); 1.9	37 (29.8%); 0.8
Bully-Victims (n = 315)	27 (20.9%); -3.2*	43 (34.1%); 0.2	190 (33.5%); 0.2	55 (44.4%); 2.8*
Not-involved (n = 305)	57 (44.2%); 3.1*	47 (37.3%); 1.3	173 (30.5%); -1.4	28 (22.6%); -2.5*

Note: Cross tabulation bullying roles by weight status. Observed frequencies (f), percentages (%; by column) and standardized residuals by chi square test (SR)

* p < .05 (standardized residuals ± 2)

### Association between weight status and different types of victimization

The results concerning the association between weight status categories, gender and the different types of victimization, accounting for age, ethnicity and parental education, are summarized in Table 2.

**Table 2:** Victimization and weight status.

	Weight Status									Gender	Gender by Weight Status
	NW	%	OW	%	Mod-OB	%	Sev-OB	%	F	F	F
Teasing for physical appearance	1.39^a^±.11	11.7	1.65^a^ ±.11	21.4	2.10^b^ ±.05	38.1	2.624^c^ ±.12	54.0	24,59***	.42	3.93**
Teasing for other reasons	1.50 ±.09	15.5	1.37 ±.09	12.8	1.46 ±.04	15.8	1.65 ±.09	23.4	1.36	.68	1.38
Name calling	1.24 ^a^ ±.10	10.1	1.62 ^b^ ±.10	20.6	1.74 ^b^ ±.05	24.7	2.15 ^c^ ±.11	44.4	14.01***	.03	0.8
Physical victimization	1.22 ^a^ ±.08	5.4	1.32 ±.08	8.7	1.41 ±.04	11.5	1.54 ^b^ ±.08	21.0	3.23*	3.38	1.91
Threatens	1.04 ^a^ ±.05	0.8	1.16 ±.05	5.6	1.18 ±.02	5.5	1.34 ^b^ ±.05	11.3	3.39*	1.85	425
Spreading rumors	1.24 ±.07	8.5	1.33 ±.07	11.9	1.36 ±.04	11.5	1.39 ±.07	14.5	1.19	.03	3.67*
Ignoring	1.17 ±.07	6.3	1.18 ±.06	6.2	1.33 ±.04	10.1	1.35 ± .07	14.5	2.54	.09	2.72
Stealing	1.40 ±.09	8.5	1.59 ±.08	20.6	1.52 ±.04	15.9	1.76 ±.09	26.6	2.20	.14	1.36
Exclusion from sports activities	1.12 ^a^ ±.07	3.9	1.24 ±.07	8.7	1.31 ±.03	10.4	1.52 ^b^ ±.07	18.5	4.48**	1.36	.08
Exclusion from group activities	1.18 ^a^ ±.08	5.4	1.33 ±.08	10.4	1.42^b^ ±.04	14.3	1.59 ^b^ ± .08	18.5	4.66**	.08	-03
Exclusion from parties	1.21 ±.07	7.8	1.25 ±.07	8.7	1.38 ±.03	12.5	1.36 ±.07	10.5	2.47	.03	.07
Total Victimization score	12.48^a^ ±.64		13.62^a, b^ ±.44		14.85^b^ ±.20		17.14^c^ ±.44		16.62***	.07	2.15

Note: Comparison between normal weight, overweight, moderate obese and severe obese participants. Adjusted means, standard errors, percentages, ANOVA’s F values, and statistical significance. Percentages indicate the numbers of subjects who declare to have been victimized 2 or 3 times a month or more. ANOVA was performed on log transformed values, but untransformed data are shown. Different letters indicate statistical differences (p < .05) between groups based on adjusted means and Sidak post-hoc test

* p < .05

** p < .01

*** p < .001

The significant main effects concerning weight status categories were found for the following types of victimization: teasing for physical appearance, name calling, physical victimization, being threatened, exclusion from sports activities and exclusion from group activities. The effect size was amplified when we considered the total victimization score obtained by summing the scores of all items. The Sidak post-hoc comparison test revealed that Sev-OB children were more frequently teased for physical appearance and by being called nasty names compared to the other groups. Sev-OB children also had higher levels of physical victimization, being threatened, exclusion from sports and group activities, compared to the NW group. Mod-OB participants were more frequently teased for physical appearance, by being called nasty names, and for exclusion from group activities, compared to OW and NW groups. Lastly, considering the total victimization score, Sev-OB children were more globally victimized than the other groups, Mod-OB were more victimized than NW, while there were not significant differences between NW and OW.

Within each weight-based category, the percentages of participants, who reported having been victimized two or three times a month or more for each type of victimization are also shown in Table 2. The percentages for all of the detected types of victimization were higher in Sev-OB compared to the other groups.

**Fig 1:**
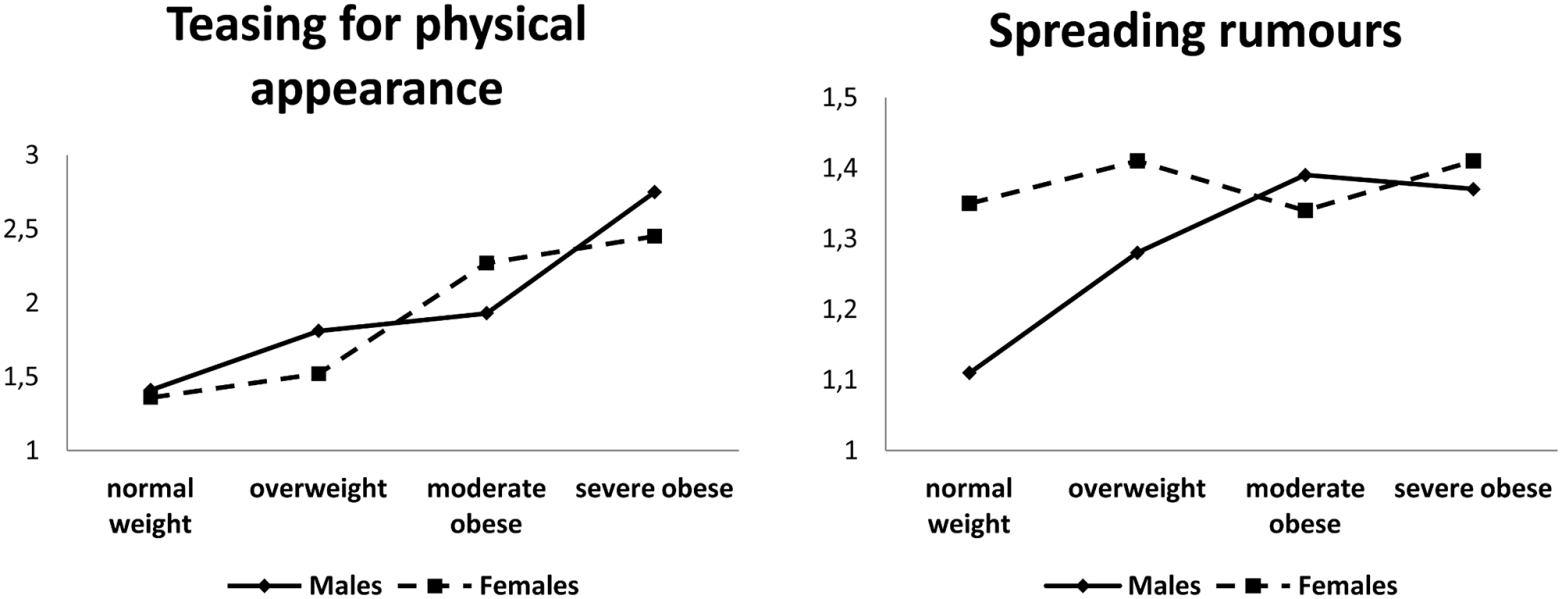
Interaction gender by weight status for victimization. The graph shows the different increase of victimization in males and females in function of the weight status.

We did not find any significant gender effect. A moderate interaction effect “gender by weight status” was found for “teasing for physical appearance” and for “spreading rumours”, because the victimization score increases more sharply in males than in females (Fig 1) in the function of the weight status. The covariate effects were very weak: Younger people suffered more from physical victimization and stealing.

### Association between weight status and different types of bullying behaviors

The results concerning the association between weight status categories, gender and the 11 different types of bullying behaviors, accounting for age, ethnicity and parental education, are summarized in Table 3.

**Table 3:** Bullying and weight status.

	Weight Status									Gender	Gender by Weight Status
	NW	%	OW	%	Mod-OB	%	Sev-OB	%	F	F	F
Teasing for physical appearance	1.24 ±.07	9.3	1.31 ±.07	9.5	1.35 ±.03	10.6	1.38 ±.06	14.5	0.87	3.57	1.99
Teasing for other reasons	1.24 ±.07	10.3	1.27 ±.07	6.4	1.29 ±.03	8.9	1.31 ±.06	10.6	0.25	2.84	2.40
Name calling	1.34 ±.08	10.2	1.38 ±.07	13.5	1.38 ±.03	11.9	1.51 ±.08	18.5	1.1	4.49*	1.54
Physical bullying	1.15 ^a^ ±.06	3.9	1.26 ±.03	7.1	1.24 ±.06	6.9	1.45 ^b^ ±.06	16.1	3.67**	6.40**	4.79**
Threatens	1.04 ^a^ ±.04	0.8	1.12 ±.04	3.2	1.08^a^ ±.02	2.3	1.23 ^b^ ±.04	8.9	4.31**	5.89	1.82
Spreading rumors	1.07 ±.04	1.6	1.16 ±.04	5.6	1.09 ±.02	2.1	1.16 ±.04	5.7	1.98	.97	2.58
Ignoring others	1.54 ±.08	18.8	1.66 ±.08	24,6	1.60 ±.04	19.1	1.58 ± .09	21.8	0.4	.06	2.58
Stealing	1.05 ±.04	2.3	1.13 ±.04	2.4	1.10 ±.02	2.8	1.14 ±.04	4.8	1.10	.72	5.54***
Exclusion from sports activities	1.21 ±.05	6.3	1.18 ±.04	3.2	1.15 ±.02	4.8	1.21 ±.05	8.1	0.7	10.33***	2.26
Exclusion from group activities	1.18 ^a^ ±.06	7.0	1.27 ±.06	8.7	1.18^a^ ±.03	5.3	1.37^b^ ± .06	13.7	2.18*	.61	4.27**
Exclusion from parties	1.20 ±.06	4.7	1.21 ±.06	7.1	1.23 ±.03	7.3	1.31 ±.06	11.3	0.68	.92	3.02
Total Bullying score	12.22^a^ ±.32		12.89 ±.31		12,62 ±.14		13.48^b^ ±.32		2.87*	5.30*	8.04***

Note: Comparison between normal weight, overweight, moderate obese and severe obese. Adjusted means, standard errors, percentages, ANOVA’s F values, and statistical significance. Percentages indicate the numbers of subjects who declare to bully others 2 or 3 times a month or more. ANOVA was performed on log transformed values, but untransformed data are shown. Different letters indicate statistical differences (p < .05) between groups based on adjusted means and Sidak post-hoc test

* p < .05

** p < .01

*** p < .001

The significant main effects concerning weight status categories were found for physical bullying, being threatened, exclusion from group activities, and the total bullying score. The Sidak post-hoc comparison test revealed that Sev-OB children showed higher scores for these items than NW children.

**Fig 2:**
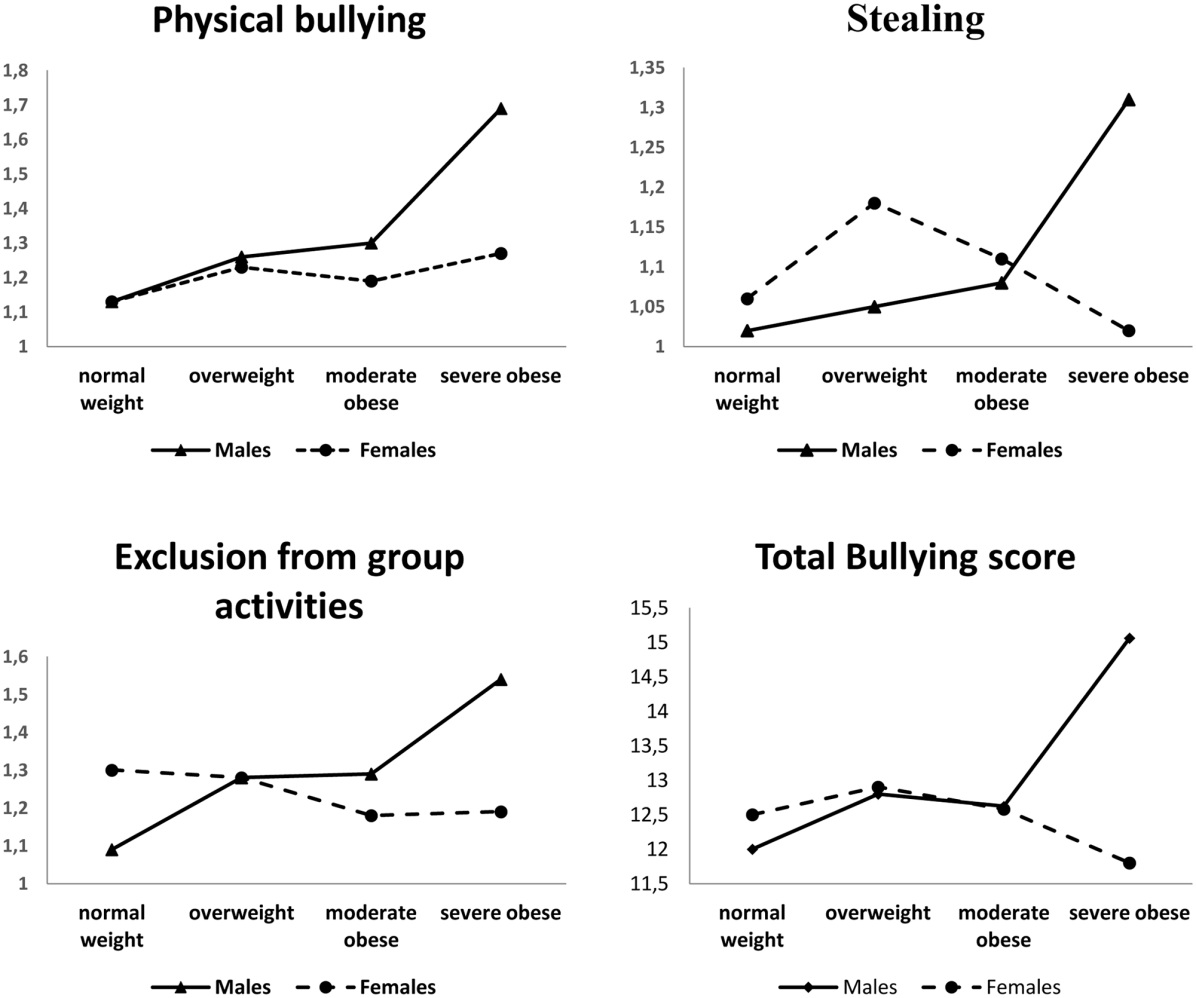
Interaction gender by weight status for bullying. The graph show the different increase of bullying in males and females in function of the weight status.

The gender effects concerned name calling, physical bullying, exclusion from sport activities and the total bullying score, as males were more involved in these behaviors than females. Significant effects were found for the interaction of weight status by gender in the following types of bullying: physical bullying, stealing, exclusion from group activities, and total bullying score. The significant interactions are shown in Fig 2. A common pattern emerged: The above-mentioned types of bullying were significantly more frequent in Sev-OB males compared to Sev-OB females.

## Discussion

Independent studies carried out on bullying or childhood obesity showed that both issues are health concerns in the Italian pediatric population [37–39]. The idea of the present study was originated by an intense debate within the Childhood Obesity Group of the Italian Society of Pediatric Endocrinology and Diabetology (ISPED) about the psycho-social consequences of overweight and obesity. Fourteen clinics for the treatment of childhood overweight-obesity, distributed throughout Italy, took part in the study, allowing us to collect a large sample of Italian weight loss-treatment seeking children and adolescents and to guarantee a shared procedure in the anthropometric and questionnaire data collection and analysis.

In the last ten years, wide research has shown that being overweight or obese is a prevalent reason why children and adolescents are teased or bullied in school or in other contexts. Nevertheless, the present study contains some elements of novelty in this field of research because: i) it investigates in depth which type of victimization or bullying is more frequent among children and adolescents exhibiting different degree of overweight up to severe obesity; ii) it’s one of the few studies [30] carried out for overweight and obese outpatient children and adolescents; and iii) it is the first systematic study conducted on this topic in Italy involving a wide pediatric population.

A first result of the present study is that Sev-OB participants were more frequently involved in the bullying episodes, than other subgroups, because only 22.6% of individuals belonging to the Sev-OB group were involved as a bully, victim or bully-victim. Consistently with the previous literature, we found that normal-weight individuals were less involved in bullying. Otherwise, the percentage of pure victims in the function of weight status does not significantly differ among the four weight subgroups. A noteworthy result is the high percentage (44.4%) of Sev-OB belonging to the dual role of bully-victim. Bully-victims are individuals who adhere, at the same time, to an active-aggressive and passive-anxious behavioral pattern; they are considered at highest risk for behavioral and psychiatric problems [40] because they show serious problems in emotional regulation. Otherwise, behavioral and emotional problems, and trouble in establishing positive relations with peers, are found in many, though not all, obese children, with a higher prevalence in clinical, treatment-seeking samples [7, 41] such as our sample. Major attention to the linkage between weight-status and bullying could highlight the origin of psychological problems in obese children.

In analyzing specific involvement in different types of bullying or victimization in the function of weight status categories, we found interesting results that provide a more comprehensive relationship between bullying and obesity. Comparing among the four weight-stratified groups, each of the 11 types of victimization and accounting for the possible confounding effects of gender, age, ethnicity and parental educational background, we found a main effect of weight status on many types of victimization. Consistent with other studies [30], teasing for physical appearance had the highest scores among obese samples. Post-hoc analyses revealed that Mod- and Sev-OB children significantly differed from the other two groups of overweight and normal-weight children, which did not statistically differ from each other. In general, we did not find differences between overweight and normal weight, except for name calling. This result is intriguing although further investigation is needed. Given the high prevalence of overweight in Italy (in some regions, 50% of children are overweight or obese children), we can argue that being overweight is considered a quasi-normative status, if not even synonymous with good health. On the other hand, the lack of difference between overweight and normal weight is not reported by other studies carried out in countries where the prevalence of overweight and obese children is as high as in Italy. The condition of Sev-OB participants was very different. In fact, compared to normal-weight these individuals showed not only the highest values in verbal victimization directly linked to their physical appearance (e.g. teasing for physical aspect) but they also significantly suffered from the highest levels of physical and relational victimization, because they were more often exposed to physical attacks, and exclusion from sports and group activities. In general, we can assert that Sev-OB participants may suffer from bad relationships with peers—at least in some settings -which may be rooted in the negative stereotypes of overweight and obesity [42] that start early in life [20, 27]. Considering that weight-based victimization is often a long-term experience [43–44], these negative experiences can have a crucial role in developing psychological and psychiatric problems. In particular, limited to specific types of victimization, males seem to be more at risk than females.

More complex is the nature of the involvement of Sev-OB children as bully-perpetrators. A different behavior emerged between genders. Bullying behavior tended to increase in males in the function of weight status, reaching a peak in Sev-OB participants, whereas it decreased in females. Further studies are needed to explain why an aggressive-reactive pattern of behavior prevails in Sev-OB males, while a passive pattern of behavior prevails in Sev-OB females. It can be hypothesized that the stigma related to severe obesity is more interiorized among females due to the overwhelming prevalence of thin and lean female imagery in the mass media in western societies and a consequent negative self-image [31] which reduces the psychological resources to react to victimization.

### Strengths and Limitations

Our investigation presents some strengths consisting in a wide sample of the overweight and obese pediatric population and in the reliability of the data collection and analysis procedures. It offers some elements of novelty in the literature consisting in the analysis of the specific types of bullying and victimization and highlighting the pervasive nature of victimization in severely obese individuals and the differences in aggressive behavior in males and females. Nevertheless, there are some limitations. First, this study is based on cross-sectional data, and therefore it is not possible to make conclusions about causality. Longitudinal research is needed to establish the correct causal pathways. Moreover, the overweight and obese participants were weight loss treatment–seeking outpatients who may perceive obesity with more seriousness than untreated obese individuals and, consequently, suffer more from psychological or physical issues than obese patients who are not in treatment. Further research in untreated obese subjects, exploring their specific self-awareness and self-image, should therefore be addressed.

### Implications

The results of the present research imply that a common policy to contrast obesity and bullying need to be enhanced. The implementation of programs to fight bullying within different settings has indicated preferences for specific strategies involving friends, peers, teachers/coaches, and lastly parents [45]. Community and school policy should be aimed at fighting prejudices and stereotypes, which can lead to bullying among peers. Although the threshold for the fatness perception has risen in recent decades given the increased overweight excess among the population, the stigma toward obese children, and in particular those who are severely obese, is still high. At the same time, school programs should be addressed to promote a healthy life style aimed at preventing overweight and obesity. Even if it is ingenuous to infer a cause-effect relation between obesity and bullying, it is certainly true that a good health condition fosters assertiveness and reduces the pretexts of teasing and exclusion from peer activities. Nevertheless, in addition to community and school policies for preventing obesity and victimization, our results highlight that obese individuals need support [45]. Health professionals who adequately acknowledge and are trained about the psychological correlates of obesity would be able to give a support to young outpatients who are upset about negative peer relationships.

## Conclusions

In conclusion, our study presents some points of novelty with respect to previous investigations about the relationship between overweight and obesity and bullying. First, it highlights the high involvement of severe obesity in the dual role of bully-victim, which has the highest psychosocial implications among the bullying roles. Second, obese participants and overall severe obese young individuals suffer from different types of victimization, specifically not only teasing for their physical appearance but also being threatened, physical victimization and exclusion, which highlights a condition of a global vulnerability. Third, severely obese male individuals seem to be at higher risk of being bullying perpetrators. Further studies are needed to confirm these results and to explain the social and psychological mechanisms that link obesity and bullying.

## References

[1] World Health Organization. WHO global strategy on diet, physical activity and health: a framework to monitor and evaluate implementation. WHO Document Production Services: Geneva, 2008.

[2] Anderson PM, Butcher KE. Childhood obesity: trends and potential causes. *Future Child* 2006; 16: 19–45. 10.1353/foc.2006.0001 16532657

[3] Griffin Smith R, Gross AM. Bullying: prevalence and the effect of age and gender. *Child Fam Behav Ther* 2006; 28: 13–37.

[4] Hong JS, Espelage DL. A review of research on bullying and peer victimization in school: An ecological system analysis. *Aggress Violent Beh* 2012; 17: 311–322.

[5] Griffiths LJ, Wolke D, Page AS, Horwood JP, ALSPAC Study Team. Obesity and bullying: different effects for boys and girls. *Arch Dis Child* 2006; 91: 121–125. 16174642 10.1136/adc.2005.072314 PMC2082670

[6] Janssen I, Craig WM, Boyce WF, Pickett W. Associations between overweight and obesity with bullying behaviors in school-aged children. *Pediatrics* 2004; 113: 1187–1194. 15121928 10.1542/peds.113.5.1187

[7] Lumeng JC, Forrest P, Appugliese DP, Kaciroti N, Corwyn RF, Bradley RH. Weight status as a predictor of being bullied in third through sixth grades. *Pediatrics* 2010; 125: e1301–e1307. 10.1542/peds.2009-0774 20439599 PMC4174570

[8] Puhl RM, Heuer CA. The Stigma of Obesity: A Review and Update. *Obesity* 2009; 17: 941–964. 10.1038/oby.2008.636 19165161

[9] Brixval CS, Rayce SLB, Rasmussen M, Holstein BE, Due P. Overweight, body image and bullying—an epidemiological study of 11- to 15-years olds. *Eur J Pub Health* 2012; 22: 126–130. 21382970 10.1093/eurpub/ckr010

[10] Puhl RM, King KM. Weight discrimination and bullying. *Best Pract Res Clin Endocrinol Metab* 2013; 27: 117–127. 10.1016/j.beem.2012.12.002 23731874

[11] Olweus D. *Bullying in school*: *what we know and what we can do*. Blackwell: Oxford, 1993.

[12] Smith PK. Bullying: recent developments. *Child Adolesc Ment Health* 2004; 9: 98–103. 10.1111/j.1475-3588.2004.00089.x 32797492

[13] Arseneault L, Bowes L, Shakoor S. Bullying victimization in youths and mental health problems: ‘Much ado about nothing’? *Psychol Med* 2010; 40: 717–729. 10.1017/S0033291709991383 19785920

[14] Hawker DS, Boulton MJ. Twenty years’ research on peer victimization and psychosocial maladjustment: a meta-analytic review of cross-sectional studies. *J Child Psychol Psychiatr* 2000; 41: 441–455. 10836674

[15] Gini G, Pozzoli T. Association between bullying and psychosomatic problems: a meta-analysis. *Pediatrics* 2009; 123:1059–1065. 10.1542/peds.2008-1215 19255040

[16] Klomek A, Marrocco F, Kleinman M, Schonfeld IS, Gould MS. Bullying, depression, and suicidality in adolescents. *J Am Acad Child Adolesc Psychiatry* 2007; 46: 40–49. 17195728 10.1097/01.chi.0000242237.84925.18

[17] Perren S, Hornung R. Bullying and delinquency in adolescence: victims' and perpetrators' family and peer relations. *Swiss J Psychol* 2005; 64: 51–64.

[18] Bacchini D, Affuso G, Trotta T. Temperament, ADHD and peer relations among school children: the mediating role of school bullying. *Aggr Behav* 2008; 34: 447–459. 10.1002/ab.20271 18512705

[19] Griffiths LJ, Page AS. The impact of weight-related victimization on peer relationships: The female adolescent perspective. *Obesity* 2008; 16: S39–S45. 10.1038/oby.2008.449 18978762

[20] Puhl RM, Latner JD. Stigma, obesity, and the health of the nation’s children. *Psychol Bull* 2007; 133: 557–580. 17592956 10.1037/0033-2909.133.4.557

[21] Puhl RM, Luedicke J. Weight-based victimization among adolescents in the school setting: Emotional reactions and coping behaviors. *J Youth Adolesc* 2012; 41: 27–40. 10.1007/s10964-011-9713-z 21918904

[22] Puhl RM, Luedicke J, Heuer C. Weight-based victimization toward overweight adolescents: observations and reactions of peers. *J Sch Health* 2011; 81: 696–703. 10.1111/j.1746-1561.2011.00646.x 21972990

[23] Krukowski RA, West DS, Philyaw Perez A, Bursac Z, Phillips MM, Raczynski JM. Overweight children, weight-based teasing and academic performance. *Int J Pediatr Obes* 2009; 4: 274–280. 10.3109/17477160902846203 19922042

[24] Bucchianeri MM, Eisenberg ME, Neumark-Sztainer D. Weightism, racism, classism, and sexism: shared forms of harassment in adolescents. *J Adolesc Health* 2013; 53: 47–53. 10.1016/j.jadohealth.2013.01.006 23566562 PMC3691304

[25] Puhl RM, Luedicke J, DePierre JA. Parental Concerns about Weight-Based Victimization in Youth. *Child Obes* 2013; 9:1–9. 24147818 10.1089/chi.2013.0064 PMC3868270

[26] Eisenberg ME, Neumark-Sztainer D, Story M. Associations of weight-based teasing and emotional well-being among adolescents. *Arch Pediatr Adolesc Med* 2003; 157: 733–738. 12912777 10.1001/archpedi.157.8.733

[27] Hayden-Wade HA, Stein RI, Ghaderi A, Saelens BE, Zabinski MF, Wilfey DE. Prevalence, characteristics, and correlates of teasing experiences among overweight children vs. non-overweight peers. *Obes Res* 2005; 1: 1381–1392. 10.1038/oby.2005.167 16129720

[28] Friedman KE, Reichmann SK, Costanzo PR, Zelli A, Ashmore JA, Musante JC. Weight stigmatization and ideological beliefs: relation to psychological functioning in obese adults. *Obes Res* 2005; 13: 907–916. 15919845 10.1038/oby.2005.105

[29] Wang J, Iannotti RJ, Luk JW. Bullying victimization among underweight and overweight U.S. youth: differential associations for boys and girls. *J Adolesc Health* 2010; 47: 99–101. 10.1016/j.jadohealth.2009.12.007 20547298 PMC2887712

[30] Puhl RM, Peterson JL, Luedicke J. Weight-based victimization: a comprehensive assessment of weight-loss treatment-seeking youth. *Pediatrics* 2013; 131: e1–e9. 10.1542/peds.2012-1106 23266918

[31] Reulbach U, Ladewig E, Nixon E, O'Moore M, Williams J, O'Dowd T. Weight, body image and bullying in nine year old children. *J Paediatr Child Health* 2013; 49: 288–293. 10.1111/jpc.12159 23530984

[32] Kukaswadia A, Craig W, Janssen I, Pickett W. Bullying as a mediator of relationships between adiposity status and weapon carrying. *Int J Public Health* 2012; 57: 505–512. 10.1007/s00038-011-0329-6 22234343

[33] Kuczmarski RJ, Ogden CL, Guo SS, Grummer-Strawn LM, Flegal KM, Mei Z, et al 2000 CDC Growth Charts for the United States: methods and development. *Vital Health Stat* 2002; 246:1–190. 12043359

[34] Solberg ME, Olweus D. Prevalence estimation of school bullying with the Olweus Bully/Victim Questionnaire. *Aggr Behav* 2003; 29: 239–268.

[35] Bacchini D, Esposito G, Affuso G. Social experience and school bullying. *J Community Appl Soc Psychol* 2009; 19: 17–32.

[36] Vieno A, Gini G, Santinello M. Different forms of bullying and their association to smoking and drinking behavior in Italian adolescents. *J School Health* 2011; 81: 393–9, 10.1111/j.1746-1561.2011.00607.x 21668879

[37] Smith PK, Morita Y, Junger-Tas J, Olweus D, Catalano R, Slee P (eds.) *The nature of school bullying*. *A cross-national perspective*. Routledge: London & New York, 1999.

[38] Cacciari E, Milani S, Balsamo A, Spada E, Bona G, Cavallo L, et al Italian cross-sectional growth charts for height, weight and BMI (2 to 20 yr). *J Endocrinol Invest* 2002; 29: 581–93. 10.1007/BF03344156 16957405

[39] Lobstein T, Frelut ML. Prevalence of overweight among children in Europe. *Obes Rev* 2003; 4: 195–200. 14649370 10.1046/j.1467-789x.2003.00116.x

[40] Wolke D, Woods S, Bloomfield L, Karstadt L. The association between physical and relational bullying and behavior problems among primary school children. *J Child Psychol Psychiatry* 2000; 41: 989–1002. 11099116

[41] Puder JJ, Munsch S. Psychological correlates of childhood obesity. *Int J Obes* 2010; 34: S37–43. 10.1038/ijo.2010.238 21151145

[42] Giletta M, Scholte RH, Engels RC, Larsen JK. Body mass index and victimization during adolescence: the mediation role of depressive symptoms and self-esteem. J. Psychosom Res 2010; 69: 541–547. 10.1016/j.jpsychores.2010.06.006 21109041

[43] Eisenberg ME, Neumark-Sztainer D, Haines J, Wall M. Weight-teasing and emotional well-being in adolescents: longitudinal findings from Project EAT. *J Adolesc Health* 2006; 38: 675–683 16730595 10.1016/j.jadohealth.2005.07.002

[44] Haines J, Hannan PJ, van den Berg P, Eisenberg ME, Neumark-Sztainer D. Weight-Related Teasing from Adolescence to Young Adulthood: Longitudinal and Secular Trends between 1999 and 2010. *Obesity* 2013; 21: e428–e434. 10.1002/oby.20092 23585224 PMC3714368

[45] Puhl RM, Peterson JL, Luedicke J. Strategies to address weight-based victimization: youths' preferred support interventions from classmates, teachers, and parents. *J Youth Adolesc* 2013; 42: 315–327. 10.1007/s10964-012-9849-5 23117953

